# Feasibility of focal cerebral ischemia and reperfusion surgery combined with chronic unpredictable mild stress to simulate the post-stroke depressive state in rats

**DOI:** 10.1186/s12993-015-0085-5

**Published:** 2015-12-24

**Authors:** Lingchuan Niu, LiXinhao Jin, Yanhong Zhang, Bing Liu, Changqing Li

**Affiliations:** Department of Neurology, The Second Affiliated Hospital of Chongqing Medical University, Linjiang Load 76, Chongqing, China

**Keywords:** Focal cerebral ischemia and reperfusion, Chronic unpredictable mild stress, Depression, Stroke

## Abstract

**Objective:**

To evaluate the feasibility of focal cerebral ischemia and reperfusion (FCIR) surgery combined chronic unpredictable mild stress (CUMS) to simulate the post-stroke depression (PSD) state in rats.

**Methods:**

Sprague–Dawley male rats were divided randomly into five groups: the normal, sham, FCIR, CUMS, and FCIR + CUMS (F/C) groups. Rats in the FCIR and F/C groups underwent an FCIR operation. Rats in CUMS and F/C groups were single-housed and exposed to CUMS for 4 weeks. Rats in the F/C group underwent CUMS for 4 weeks after FCIR surgery. The gain in bodyweight, the sugar consumption ratio in a sucrose preference test (SPT), and behavior, including spontaneous moves (SM), the duration of time spent in the center arena (duration), and the number of rearings (rearing) in an open field test (OFT), were evaluated.

**Results:**

Rats in the CUMS and F/C groups had a smaller gain in bodyweight (P < 0.05). The sugar consumption ratio was reduced significantly in the CUMS and F/C groups compared with the normal and FCIR groups (P < 0.05). The number of SM was significantly lower in the FCIR group compared with the normal group. SM, duration, and rearing were reduced significantly in the CUMS and F/C groups relative to the normal group. Furthermore, the number of rearings was lower in the F/C group compared with the CUMS group.

**Conclusion:**

Anhedonia, a lack of curiosity, and inactivity were observed in the F/C rats, which exhibited depression-like symptoms after FCIR surgery.

## Background

Depression is a very common neuropsychiatric sequel in the post-stroke population, affecting approximately 30–50 % of patients in the first year [1–3]. It occurs most frequently in the first few months after stroke, but an additional incidence peak might occur 2–3 years after stroke [4]. Post-stroke depression (PSD) leads to a poor response to rehabilitation, a slow physical recovery, impaired quality of life, and increased mortality. The treatment and mechanism of PSD has been a hot area for research in recent years. However, it is unclear how to establish an animal model of PSD. Although the ideal model must characterize the depression caused directly by stroke, the proportion of spontaneous depression after stroke is very low in rats. In a more recent method single-housed rats were exposed to chronic unpredictable mild stress (CUMS) to establish an animal model of depression.

The aim of the current study was to find a suitable method of simulating the PSD state in rats for further research into PSD. First, the rats underwent a focal cerebral ischemia and reperfusion (FCIR) operation according to the modified Longa nylon method. Then, rats with neurological symptoms according to the modified neurological severity score (mNNS) [5] were selected and exposed to CUMS while being single housed for 4 weeks. The bodyweight of the rats was measured, and alterations in physiological behavior were determined using sucrose preference test (SPTs) and open field tests (OFTs).

## Methods

### Animals and treatments

Adult 13-week-old Sprague–Dawley (SD) male rats weighing 230–250 g were purchased from the Experimental Animal Center of Chongqing Medical University. The Laboratory Animal Management Committee of Chongqing Medical University authorized the experimental protocol. All animal procedures were carried out according to the guidelines of the China Animal Protection Law and were approved of by the Institutional Ethics Committee of Chongqing Medical University [Permit No. SCXK (Chongqing) 2007–0001] and the State Science and Technology Commission of China.

The rats were group housed (five per cage) in quiet in a room that was maintained at 21–22 °C with 50 % relative humidity, with 12/12-h light/dark cycles. They were allowed free access to food and water and a 1-week adaptive feed before the baseline SPT and OFT characteristics were measured. Then, 98 rats with similar baseline characteristics were selected and randomized into five groups: normal, sham, FCIR, CUMS, and F/C. Rats in the FCIR group underwent an FCIR operation. The rats in CUMS group were single-housed and exposed to CUMS for 4 weeks. Rats in F/C group underwent FCIR surgery and were then single-house while experiencing CUMS for 4 weeks. The model was created successfully in 75 rats: 10 in the normal group, 10 in the sham group, 18 in the FCIR group, 20 in the CUMS group, and 17 in the F/C group. Eight rats were excluded because of they had no neurological symptoms according to the mNNS and fifteen rats died during this research.

### Focal cerebral ischemia and reperfusion (FCIR) surgery

Focal cerebral ischemia surgery was performed according to the intraluminal occlusion technique described previously by Koizumi et al. [6]. Briefly, rats were anesthetized using 3.5 % chloral hydrate (350 mg/kg). Then, the right common carotid artery (CCA), the right external carotid artery (ECA), and the internal carotid artery (ICA) were surgically exposed via a midline incision. The CCA was ligated distally and the ECA was ligated proximally to the bifurcation of the ICA and the ECA. A nylon filament (diameter 0.24–0.26 mm) was gently advanced from the ECA into the lumen of the ICA. After 2 h of ischemia the nylon filament was carefully removed to establish reperfusion. The body temperature was maintained at 37 °C using a heating pad during the surgery. In sham group, the embolus was inserted to a distance of 12 mm and was pulled out immediately.

The neurological function of the rats was evaluated on the first day after surgery using the mNSS. The mNSS is a composite of motor (muscle status, abnormal movement), sensory (visual, tactile, and proprioceptive), beam balance, and reflex (pinna, corneal and startle reflexes) tests, and is graded on a scale of 0–18 (normal, 0; maximum deficit, 18). Two observers blinded to the experimental groupings performed all tests. The mNSS of all rats was 0 before surgery, and any rats with a score of 0 on the first day after surgery were excluded from the experiment.

### Chronic unpredictable mild stress (CUMS)

The CUMS procedure was performed as described previously [7, 8]. However, some adjustments were made to increase the unpredictability. The rats were subjected to CUMS for 28 days, including ice water swimming at 4 °C for 5 min, hot water swimming at 45 °C for 5 min, shaking once per second for 5 min, restricted access to water for 3-h following 20-h of water deprivation, tail pinch for 1 min, restricted access to food for 3-h following 20-h of food deprivation, cage tilt at 45° for 24 h, housing in a wet cage for 24-h (containing 100 g of sawdust in 200 ml water), shocking the feet at 50 mV (three times/min each for 10 s for 10 min), stroboscopic lighting for 36-h (300 flashes/min), intermittent white noise (150 dB once per min for 20 s for a total of 100 min), and an uncomfortable smell (prednisone) for 12 h. Rats were exposed to one of these 12 stimuli randomly per day, and the same stressor was not used on consecutive days.

### Behavioral tests

The rats were weighed on days 1, 29, and 57 of the study. The SPT and OFT were carried out in sequence on day 1 to obtain baseline measurements. These tests were repeated on days 22 (3rd week), 29 (4th week), and 57 (8th week) after surgery.

### Sucrose preference test (SPT)

Rats were allowed to choose between two bottles after 20-h of food and water deprivation (from 12.00 to 8.00): one contained 1 % sucrose water and the other had normal drinking water. The consumption of water and sucrose solution was estimated by weighing the bottles after 2 h. The data recorded from two bottles were analyzed for its sugar consumption ratio as its changes in flavor preference. The sucrose preference (SP) = sucrose intake water (g)/total intake water (g) × 100 %.

### Open field test (OFT)

The experimental device consisted of an open field reaction tank and an automatic data acquisition and processing system (Shanghai Xin-ruan Information Technology Co., China). The tank was sized 100 × 100 × 40 cm size, and the bottom of the box was divided into 25 equilateral squares sized 4 × 4 cm. A digital camera was placed above the box that could cover the entire field. The box was cleaned thoroughly before each animal was tested. The animal was replaced in its home cage immediately after the test.

The rats was placed in the open field reaction tank for 5 min, and its’ movement recorded using a digital camera. The number of spontaneous moves (SM), the percentage of duration time spent in the center square (duration = time spent in the center square (s)/total time (s) × 100 %), and the number of rearings (rearing) were analyzed automatically using sports track in XR-Xmaze supermaze software (Shanghai Xin-ruan Information Technology Co.).

### Statistical analysis

Data are expressed as mean ± standard error of the mean (SEM), and statistical differences between groups were compared using two-way analysis of variance (ANOVA) with time and groups as between subject factors. A value of P < 0.05 was considered statistically significant. All statistical analyses were performed using SPSS, version 17.0 (Chicago, IL, USA).

## Results

### Bodyweight

**Fig. 1:**
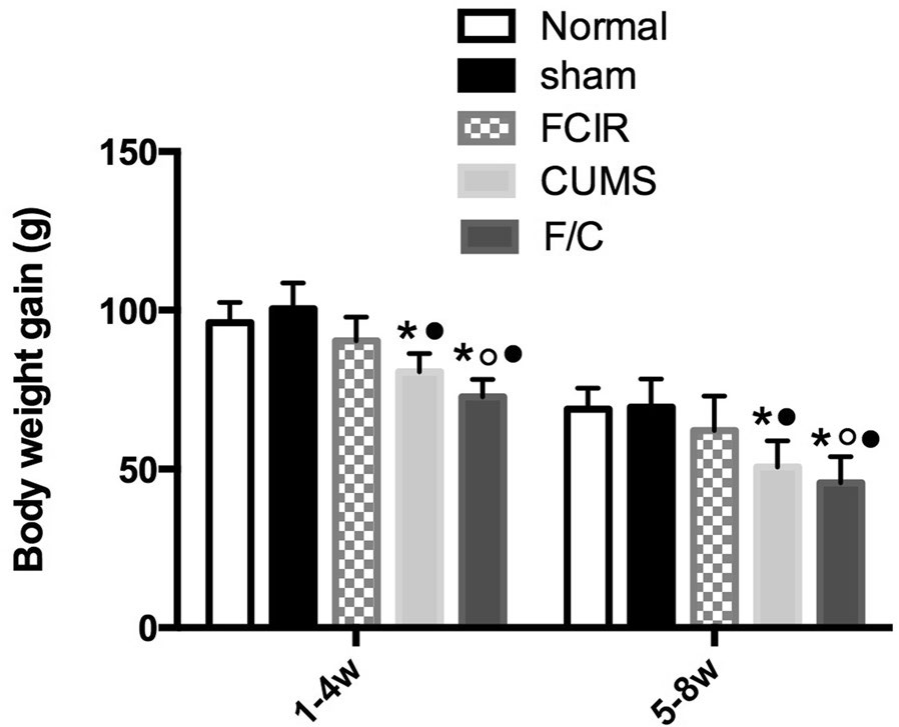
Mean gain in bodyweight at weeks 1–4 and 5–8 week in rats in the normal, sham, FCIR, CUMS, and F/C groups. ^★^P < 0.05 vs. normal rats, ^○^P < 0.05 vs. sham rats, ^●^P < 0.05 vs. FCIR rats. *SEM* standard error of the mean; *FCIR* focal cerebral ischemia and reperfusion; *CUMS* chronic unpredictable mild stress; *F/C* FCIR/CUMS

As shown in Fig. 1, the gain in bodyweight in the normal group in the first 4-week period (weeks 1–4) and the next 4-week period (weeks 5–8) was 96.4 ± 9.1 g and 68.5 ± 8.4 g, respectively. There was no significant difference between the normal and sham groups. Although there was a small decrease in bodyweight in the FCIR group compared with the normal group, the different was not statistically significant (P > 0.05). The bodyweight of rats in the CUMS and F/C groups was reduced significantly compared with the control group (P < 0.05). Finally, weight was reduced significantly in the F/C group compared with the FCIR group (P < 0.05).

### Sucrose preference test

**Fig. 2:**
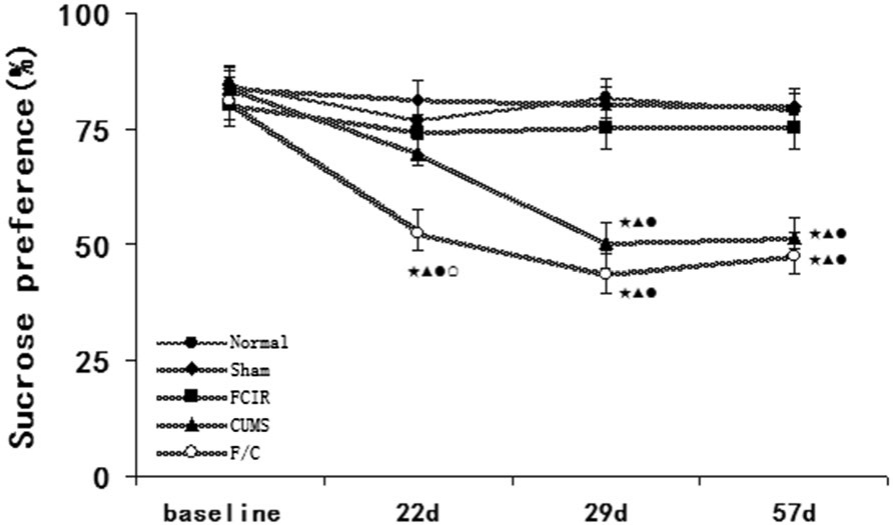
Mean SP in the normal, sham, FCIR, CUMS, and F/C groups. ^★^P < 0.05 vs. normal rats, ^▲^P < 0.05 vs. sham rats, ^●^P < 0.05 vs. FCIR rats, ^○^P < 0.05 vs. CUMS rats. *SEM* standard error of the mean; *FCIR* focal cerebral ischemia and reperfusion; *CUMS* chronic unpredictable mild stress; *F/C* FCIR/CUMS

As shown in Fig. 2, there were no significant differences in SP among the normal, sham, and FCIR groups at any point during the experiment (P > 0.05). However, SP was significantly lower in the CUMS and F/C groups compared with the normal and FCIR groups on weeks 4 and 8 (P < 0.05). Furthermore, SP was lower in the F/C group than the CUMS group on week 3 (P < 0.05).

### Open field test (OFT)

**Fig. 3:**
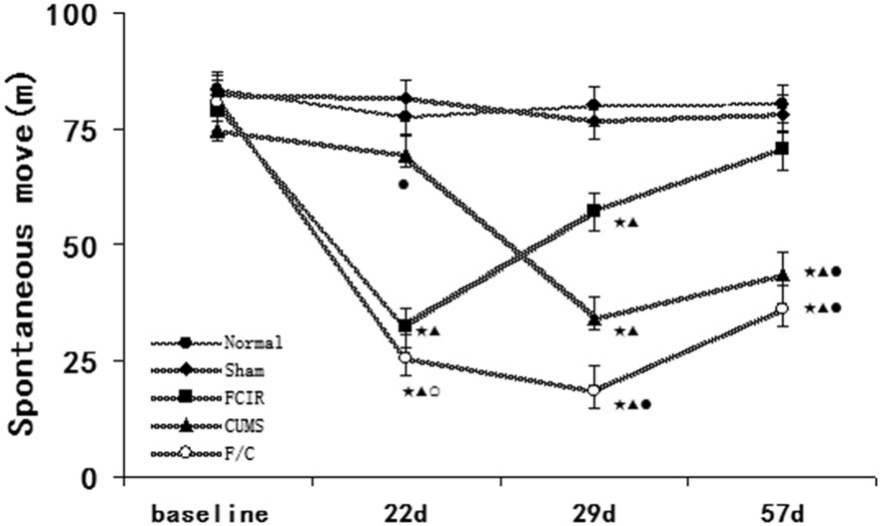
Mean SM in the normal, sham, FCIR, CUMS, and F/C groups. ^★^P < 0.05 vs. normal rats; ^▲^P < 0.05 vs. sham rats; ^●^P < 0.05 vs. FCIR rats; ^○^P < 0.05 vs. CUMS rats. *SEM* standard error of the mean; *FCIR* focal cerebral ischemia and reperfusion; *CUMS* chronic unpredictable mild stress; *F/C* FCIR/CUMS

**Fig. 4:**
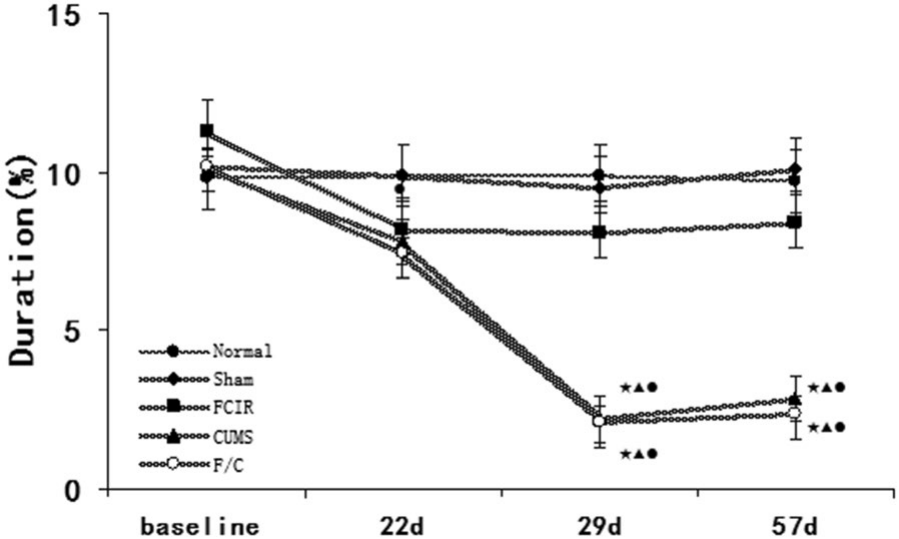
Mean percentage of duration time spent in the *center square* in the normal, sham, FCIR, CUMS, and F/C groups. ^★^P < 0.05 vs. normal rats; ^▲^P < 0.05 vs. sham rats; ^●^P < 0.05 vs. FCIR rats; ^○^P < 0.05 vs. CUMS rats. *SEM* standard error of the mean; *FCIR* focal cerebral ischemia and reperfusion; *CUMS* chronic unpredictable mild stress; *F/C* FCIR/CUMS

**Fig. 5:**
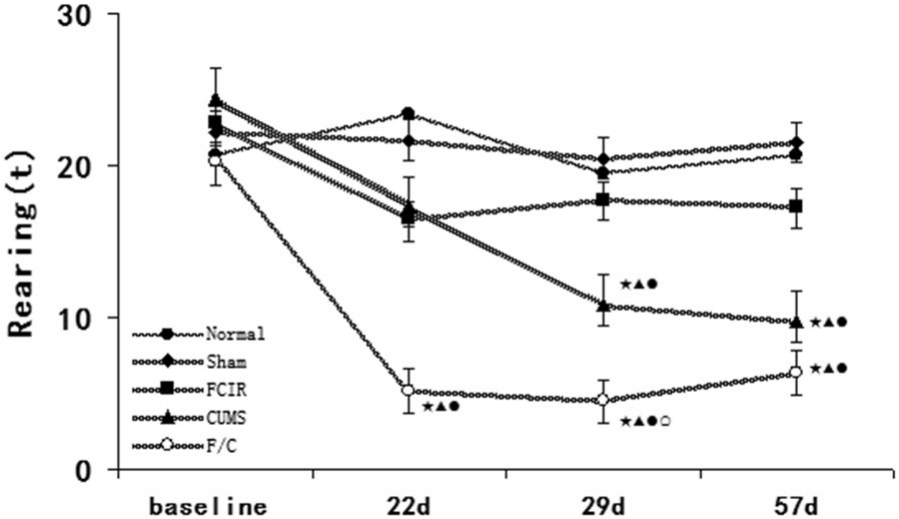
Mean number of rearings in the normal, sham, FCIR, CUMS, and F/C groups. ^★^P < 0.05 vs. normal rats; ^▲^P < 0.05 vs. sham rats; ^●^P < 0.05 vs. FCIR rats; ^○^P < 0.05 vs. CUMS rats. *SEM* standard error of the mean; *FCIR* focal cerebral ischemia and reperfusion; *CUMS* chronic unpredictable mild stress; *F/C* FCIR/CUMS

There were no significant differences between the normal and sham groups, confirming that the operation did not affect the results. As shown in Figs. 3, 4, 5, SM was significantly lower in the FCIR group than the normal group at weeks 3 and 4 (P < 0.05), but there were no significant differences in duration and rearing between the normal and FCIR groups (P > 0.05). In addition, rats in the CUMS group exhibited decreased SM, duration, and rearing compared with the normal group at weeks 4 and 8 (P < 0.05).

SM was significantly lower in the F/C group compared with the normal group on weeks 3, 4, and 8, and compared with the FCIR group on weeks 4 and 8. Finally, SM was lower in the F/C group than the CUMS group on weeks 3 and 4 (P < 0.05; Fig. 3). Duration was lower in the F/C group compared with the normal and FCIR groups on weeks 4 and 8 (P < 0.05; Fig. 4). Rearing was lower in the F/C group than the normal and FCIR groups on weeks 3, 4, and 8, and compared with the CUMS group on weeks 3 and 4 (P < 0.05; Fig. 5).

## Discussion

In the last 30 years several studies have improved the methods used to establish depression models in rats [7, 8]. CUMS is now the most widely used method of establishing a depression model in rats. However, the effectiveness of CUMS is sometimes difficult to determine, and unique strain sensitivities is a limiting factor. The current study used 12 mild stressors, including ice water swimming, hot water swimming, and shaking. Previous studies demonstrated that the use of stroboscopic lighting for 36 h causes sleep abnormalities [9, 10], and can also decrease exploratory and sexual behavior. The purpose of using 12 mild stressors in an unpredictable manner was to avoid habituation and ensure that the stimulation elicited the appropriate depression-like responses.

Psychological evaluations cannot be performed directly in rats, and models are evaluated using only behavioral observations. For example, the most common behavioral outcome measurements are SPT, OFT, forced swimming tests (FST), and tail suspension tests (TST) [11–13]. However, it remains controversial whether FST and TST are valid indicators of depression-like responses to the CUMS model. Some scholars believe that the motionless state of the animal in the FST and TST could be simply an adaptation or a fatigue phenomenon rather than true despair behavior [14, 15]. DSM-V demonstrated that anhedonia, a loss of responsiveness to pleasant events or an index of reward sensitivity, is a core symptom of depression. The most common method of assessing the anhedonia that arises following CUMS exposure in rats is SPT, which measures sugar consumption ratio as a flavor preference [16–18]. OFTs were used in the current study to evaluate exploratory and anxiety-like behavior. Other symptoms associated with depression, such as decreased libido, aggression, self care behavior, and sleep structure changes, cannot be reversed using classical clinically effective antidepressant treatment in the short term and so are rarely used in studies of PSD. Therefore, we selected SPT and OFT as the assessment methods in the current study.

Compared with rats in the baseline and normal groups, those in the CUMS group exhibited a significantly decreased gain in bodyweight, SP, and OFT (including SM, duration, and rearing) after the first 4 weeks, which is consistent with previous studies [19–22]. At the end of the next 4 weeks, the bodyweight gain and outcome of the behavioral tests indicated that the rats were still in a depressed state. This suggests that the current experimental paradigm could elicit the appropriate stress responses following CUMS exposure in rats.

Although there were no significant differences in SP, duration, and rearing during an OFT between the normal and FCIR groups, FCIR caused a significant decrease in SM on weeks 3 and 4. We hypothesize that this could be related to the movement disorders caused by the FCIR surgery. Furthermore, the SM returned to normal at week 8 because of a certain degree of recovered motor function. These results suggest that surgery might reduce some results on behalf of exploration, but it cannot cause other core symptoms of depression such as anhedonia. Therefore, FCIR surgery alone cannot completely induce depression-like responses in the same manner as CUMS.

According to a study by Burgado [23] animals began to express an increase in depressive-like behavior in the SPT after 2 weeks of predatory stress. Willner et al. found that preference deficits took at least 2 weeks to develop and were maintained for more than 2 weeks after termination of the stress regime [24]. Furthermore, some studies have shown that approximately 20 % of rats do not reduce their sucrose intake following the application of CUMS [25–28]. In the current study the sugar consumption ratio was decreased significantly in the CUMS group after 4 weeks, and this decrease was maintained for at least 4 weeks. It is possible that this might be related to the different stress intensity during CUMS. In addition, different rat strains have different sensitivity to sugar water, and the method used to detect SPT was improved. The rats in the F/C group experienced a more rapid and serious reduction in their sugar consumption ratio. This could be because the combination of the FCIR operation and CUMS decreased SPT after just 3 weeks and was retained for the next 5 weeks.

Compared with the FCIR group, rats in the F/C rats exhibited a decreased OFT on days 29 and 57. Furthermore compared with the CUMS group, rats in the F/C groups exhibited a decreased SM and rearing at certain time points. This suggests that rats that underwent F/C exhibited more serious depressive behavior compared with those that experienced only FCIR or CUMS. In addition, the current study revealed that FCIR surgery lead to anhedonia or behavioral despair in individual rats. Since the ideal rat model of PSD (depression caused directly by stroke) is very difficult to obtain, these F/C rats that exhibit neurological dysfunction and depressive behavior is a compromise for further studies.

The results of this study verify the feasibility of FCIR surgery combined with CUMS for simulating the post-stroke depressive state in rats. Moreover, the depressive behavior in F/C rats persisted at least for 4 weeks, which is an appropriate period of time for interventions and treatments. Nevertheless, since F/C is not the ideal model additional studies need to be conducted to better simulate the pathological and physiological aspects of the PSD state.

## Abbreviations

CCA: right common carotid artery
CUMS: chronic unpredictable mild stress
EC: right external carotid artery
F/C: FCIR/CUMS
FCIR: focal cerebral ischemia and reperfusion
ICA: internal carotid artery
OFT: open field test
SM: spontaneous move
SPT: sucrose preference test
SP: sucrose preference
PSD: post-stroke depression
