# Erratum to: Feasibility of focal cerebral ischemia and reperfusion surgery combined with chronic unpredictable mild stress to simulate the post-stroke depressive state in rats

**DOI:** 10.1186/s12993-016-0088-x

**Published:** 2016-01-22

**Authors:** Lingchuan Niu, Xinhao Jin, Yanhong Zhang, Bing Liu, Changqing Li

**Affiliations:** Department of Neurology, The Second Affiliated Hospital of Chongqing Medical University, Chongqing, China

## Erratum to: Behav Brain Funct (2015) 11:39 DOI 10.1186/s12993-015-0085-5

Unfortunately the original version of this article [1] contained an error. After publication it was brought to our attention that one of the authors’ names was incorrectly spelt. The original spelling reads as ‘LiXinhao Jin’, however the correct spelling should be Xinhao Jin. This has now been updated on the website.
